# Rapid Synthesis of Carbon Dots by Hydrothermal Treatment of Lignin

**DOI:** 10.3390/ma9030184

**Published:** 2016-03-09

**Authors:** Wenxin Chen, Chaofan Hu, Yunhua Yang, Jianghu Cui, Yingliang Liu

**Affiliations:** 1 Department of Chemistry, Jinan University, Guangzhou 510632, China; tchenwenxin@jnu.edu.cn; 2 College of Mechanics, Taiyuan University of Technology, Taiyuan 030024, China; huchaofan@tyut.edu.cn; 3 Guangdong Institute of Microbiology, State Key Laboratory of Applied Microbiology Southern China, Guangdong Provincial Key Laboratory of Microbial Culture Collection and Application, Guangzhou 510070, China; yangyh@gdim.cn; 4 Department of Applied Chemistry, College of Science, South China Agricultural University, Guangzhou 510642, China; jianghucui@soil.gd.cn

**Keywords:** carbon dots, lignin, H_2_O_2_, hydrothermal synthesis, bioimaging

## Abstract

A rapid approach has been developed for the fluorescent carbon dots (CDs) by the hydrothermal treatment of lignin in the presence of H_2_O_2_. The as-synthesized CDs were found to emit blue photoluminescence with excellent photostability. Moreover, the CDs displayed biocompatibility, low cytotoxicity, and high water solubility properties. Finally, the as-resulted CDs were demonstrated to be excellent probes for bioimaging and biosensing applications.

## 1. Introduction

Carbon dots (CDs) are an interesting class of carbon nanoparticles, which are being investigated for various applications due to their favorable optical stability, low toxicity, biocompatibility, and ease of functionalization [1,2,3]. Many researchers have studied the synthesis methods and photoluminescence properties of CDs. A variety of synthesis approaches such as laser ablation, electrochemical exfoliation, pyrolysis, incomplete combustion oxidation, acidic oxidation, hydrothermal treatments and microwave synthesis have been developed to prepare CDs [4,5,6,7,8,9,10,11]. Various raw material including graphite oxide, citric acid, glycerol, coffee grounds, soy milk, grass and egg have been used in the synthesis of CDs [12,13,14,15,16]. However, it is still desirable to rapidly synthesize high-quality CDs by an easy and environmentally benign method with low-cost and readily available starting materials. One such suitable raw material is lignin, which is an abundant natural organic polymer, and an excellent source of carbon. There is an increasing interest in using lignin to prepare new carbon-based materials [17,18,19]. However, it is quite difficult to degrade lignin and its derivatives due to strong carbon to carbon linkages in their molecular structure. Amongst various techniques, the hydrothermal carbonization process is a promising approach for the synthesis of novel carbon-based materials, especially CDs [20].

Herein, we report a rapid route to synthesize highly luminescent CDs by the hydrothermal treatment of lignin with the assistance of H_2_O_2_. It is well-known that H_2_O_2_ can be dissociated into hydroxyl radicals (·OH) under the photoassisted catalysis Fe^3+^/Fe^2+^ in water, and the resulting OH radical is an extremely powerful oxidizing species [21]. The synthesis approach was simple and environmentally friendly. It was demonstrated that the as-prepared CDs exhibit good luminescence property, good water solubility, narrow particle size distribution and low cytotoxicity. The CDs also showed excellent bioimaging capabilities in Hela cells. This work provides a new approach for the preparation of CDs from natural materials, and also demonstrates the potential of CDs in bio-imaging applications.

## 2. Results and Discussion

**Figure 1:**
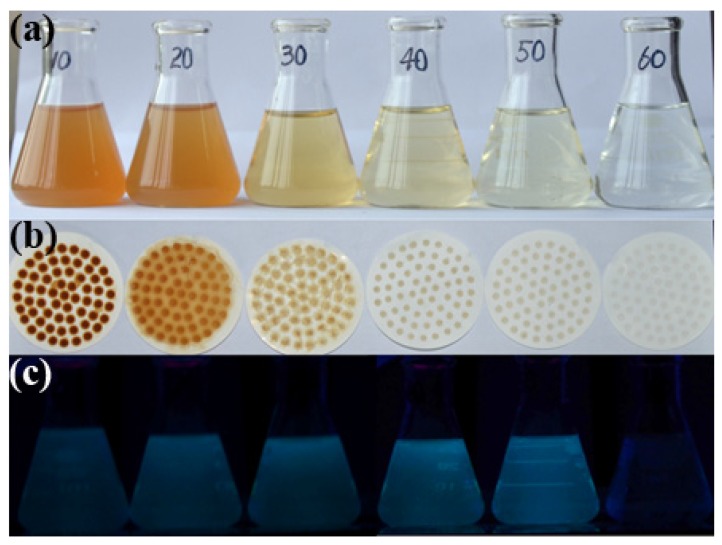
Pyrolysis results for (**a**) the initial product solution; and (**b**) the residual lignin after 10, 20, 30, 40, 50 and 60 min hydrothermal reaction; and (**c**) the corresponding filtrate image under 365 nm UV lamp irradiation.

Figure 1a indicates the pyrolysis products of lignin at 10, 20, 30, 40, 50 and 60 min, respectively. It could be seen that the color of the initial product solution became pale as the time increased. The residual lignin was retained on the filter paper (Figure 1b). The synthesis yields of the CDs were 12.06%, 10.2%, 6.67%, 2.3%, 1.05%, 0.8% corresponding to 10, 20, 30, 40, 50 and 60 min, respectively. The optical images of the CDs solution exhibited blue luminescence under UV light excitation (Figure 1c) and the pyrolysis time of 40 min exhibited the maximum brightness. The CDs solution remains transparent for half a year without precipitation.

**Figure 2:**
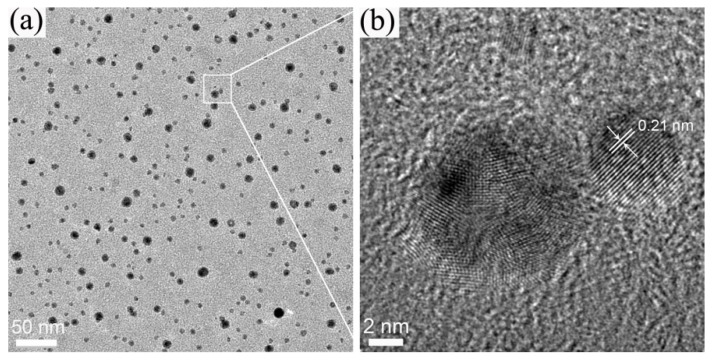
(**a**) Transmission electron microscopy (TEM); and (**b**) high-resolution TEM (HRTEM) images of the carbon dots (CDs) prepared by hydrothermal treatment of lignin.

Transmission electron microscopy (TEM) and high-resolution TEM (HRTEM) performed on the CDs, and the results are shown in Figure 2. The size of the CDs ranged from 2 to 10 nm. The HRTEM images indicated that the carbon dots have crystalline structure and the lattice spacing distance was about 0.21 nm, close to that of the graphite (100) plane [22].

**Figure 3:**
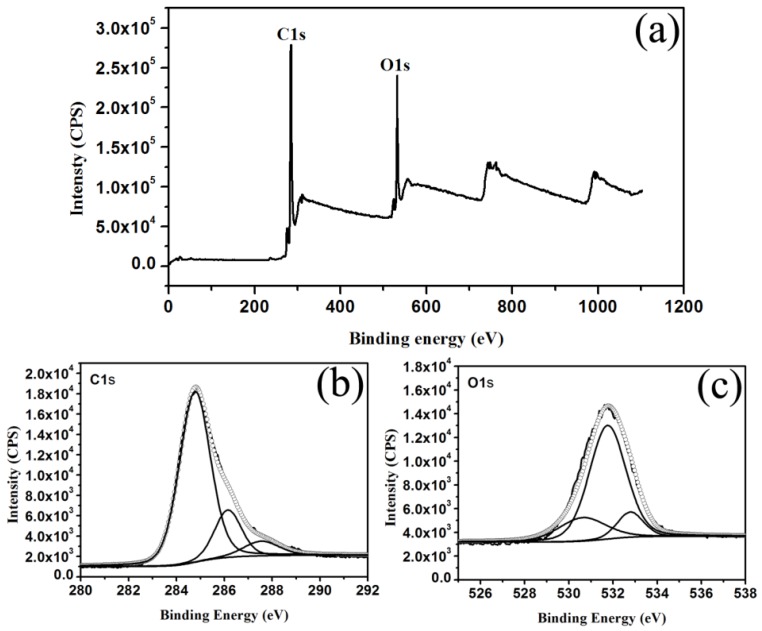
(**a**) X-ray photo-electron spectroscopy (XPS); (**b**) C_1s_; and (**c**) O_1s_ spectra of the as-prepared CDs.

From the wide-scan X-ray photo-electron spectroscopy (XPS) spectrum depicted in Figure 3, two strong peaks at 285.5 and 532.0 eV were attributed to oxygen and carbon, respectively. The elemental components of the as-prepared CDs were C (82.58%) and O (17.42%). The deconvoluted C_1s_ spectrum (Figure 3b) showed three components, which could be assigned as graphite (sp^2^) carbon at ~283.2 eV, sp^3^ carbon at ~286.1 eV, and carboxyl carbon at 288.6 eV. The O_1s_ spectrum (Figure 3c) exhibited three peaks at 530.6, 532.1 and 533.2 eV, which were attributed to the C=O, C-OH and C-O-C groups, respectively [23,24]. 

**Figure 4:**
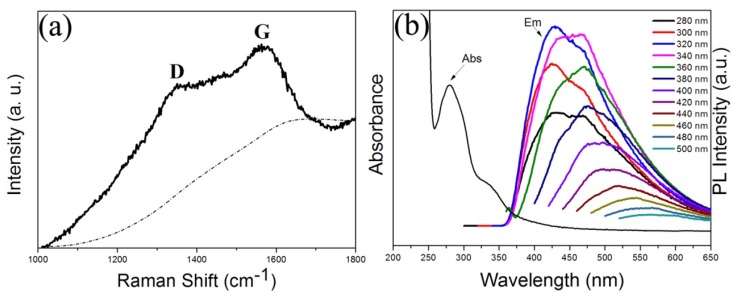
(**a**) Raman absorbance and the background is given by the dashed line; (**b**) photoluminescence (PL) spectra of the CDs at different excitation wavelengths.

**Figure 5:**
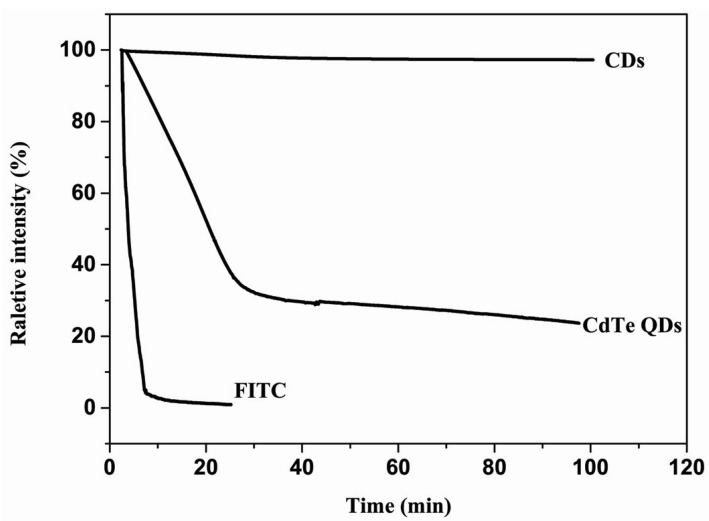
Photostability comparison of the fluorescent CDs, CdTe QDs and fluorescein isothiocyanate (FITC) in a fluorescence spectrophotometer with a 150 W Xe lamp under 360 nm excitation.

The CDs obtained after 40 min pyrolysis were characterized by Raman spectroscopy, as shown in Figure 4a. Their Raman spectrum showed a strong D band at 1382 cm^−1^, which corresponds to the sp^3^ defects in CDs. Also, a G band was observed at 1578 cm^−1^, which matched well with the disordered carbon and the sp^2^ clusters, indicating that there were aromatic groups inside CDs. It was observed that the CDs have an *I_D_/I_G_* ration of 0.91, which might be due to oxygen-rich edges of the CDs. The UV-Vis spectra of aqueous solution of CDs showed two peaks at 282 and 348 nm, indicating that there were different surface states present in the CDs solution. The fluorescence spectra of the CDs_40min_ were measured with an F-4500 fluorescence spectrometer (HITACHI, Tokyo, Japan), with a slit width of 10 nm for both excitation and emission beams. The excitation wavelength was varied from 280 to 500 nm, in 20 nm increments. The corresponding spectra are given in Figure 4b. Bright and colorful photoluminescence (PL) emissions were observed from the CDs. The emission maxima shifted as the excitation wavelength increased and exhibited a maximum PL intensity at an excitation wavelength of 320 nm and emission wavelength of 430 nm. The CDs showed excellent photostability as the fluorescence intensity did not change, even after continuous excitation under a 150 W Xenon lamp. As shown in Figure 5, the fluorescence of the fluorescein isothiocyanate (FITC) was quickly quenched within several minutes excitation and the CdTe quantum dots (QDs) were preserving 25% of the original PL intensity after 20 min excitation. The PL intensity of the CDs that we synthesized retaining 95% of the initial intensity under *ca.* 100 min excitation. The result indicated that the PL of the CDs was much more stable than of the fluorescent FITC and the CdTe QDs. We considered that the formation of CDs and their surface functionalization take place simultaneously during the hydrothermal carbonization process. The presence of large number of carboxylic acids introduces several different surface defects. These defects behave as excitation energy traps, and are responsible for the different photoluminescence behaviors. In fact, several mechanisms have been proposed to explain these unique PL properties, such as the size distribution of the CDs, a distribution of different emissive trap sites and the formation of several different polyaromatic fluorophores within the carbogenic core. However, the exact mechanism of the CDs’ PL behavior is still unclear and further studies are required to understand this property in depth. 

**Figure 6:**
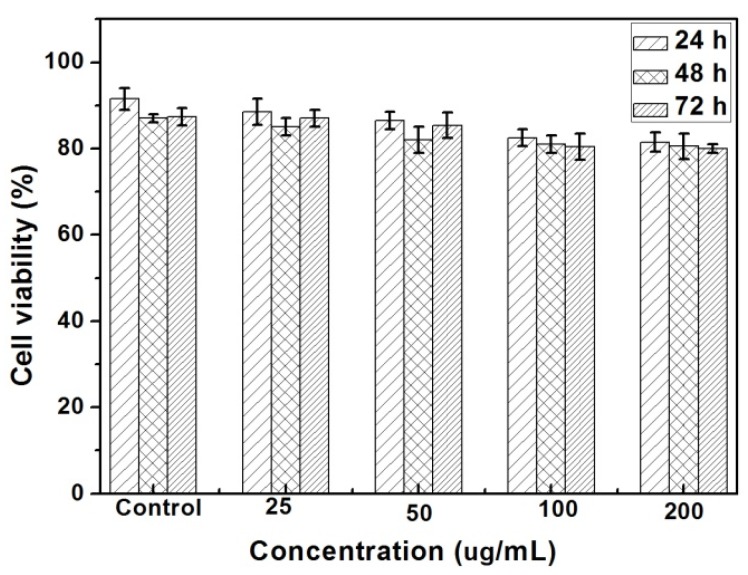
Cytotoxicity evaluations test of A549 cells with different concentrations of CDs_40min_ after 24, 48 and 72 h incubation.

To investigate the feasibility of using CDs for bio-imaging, A549 human lung adenocarcinoma cells were used to evaluate the cytocompatibility of the CDs. The cell viability of the CDs was determined by a methylthiazoleterazolium (MTT) assay. As can be seen in Figure 6, the MTT assays of cell viability reports indicate that the CDs have very low cytotoxicity. This result confirms that CDs can be used for imaging or other biomedical applications.

**Figure 7:**
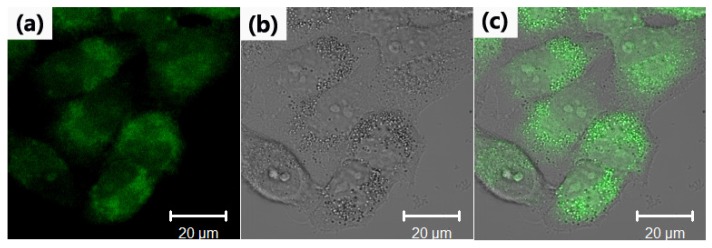
(**a**) A confocal fluorescence microphotograph of Hela cells labeled with the CDs (*λ*ex: 405 nm); (**b**) A bright field microphotograph of the cells; (**c**) An overlay image of (**a**) and (**b**).

The obtained CDs from 40 min hydrothermal carbonization were introduced into the Hela cells, and their bio-imaging capabilities were evaluated using *in vitro* confocal microscopy test. The results showed that the photoluminescent spots were observed only in the cell membrane and cytoplasmic area of the cell, indicating that the CDs were able to easily penetrate into the cell (Figure 7). This observation was is in agreement with previous studies on the interaction of living cells with nanomaterials [25]. The results illustrate that CDs can be used as fluorescence probe for bio-imaging applications. 

## 3. Materials and Methods 

### 3.1. Preparation of the Fluorescent CDs

In a typical procedure, fluorescent CDs were synthesized as follows: 100 mg lignin was dispersed in 30 mL purified water and ultrasonicated for 10 min, then 2 mL H_2_O_2_ was added, and the mixture was sealed into a 50 mL Teflon lined stainless steel autoclave, which was then placed in a muffle furnace followed by hydrothermal treatment at 180 °C for 10, 20, 30, 40, 50 and 60 min. After the reaction, the autoclave was cooled down naturally, and the obtained yellow solution was filtered with a 0.22 µm membrane filter (Millipore, Boston, MA, USA) to remove the unreacted lignin. The filtrate was subjected to dialysis for 2 days using a 3500 Da dialysis membrane (Spectrumlabs, Rancho Dominguez, CA, USA) to remove the excess H_2_O_2_. The resulting yellow solution was freeze-dried to obtain the final CDs.

### 3.2. Characterization Methods

Morphological features of the CDs were using a transmission electron microscopy (TEM, Philips TECNAI 10, Amsterdam, Holland) and field emission electron microscope (JEOL JEM-2100F, JEOL, Tokyo, Japan). X-ray photo-electron spectroscopy (XPS, AXIS ULTRA DLD, Kratos, Manchester, British) was used to investigate the functional groups present on the surface of the CDs. The fluorescence spectra of the CDs were measured with a fluorescence spectrometer (F-4500, HITACHI, Tokyo, Japan), with a slit width of 10 nm and 10 nm for excitation and emission, respectively. The excitation wavelength increased by a 20 nm increment starting from 280 to 500 nm.

### 3.3. Fluorescence Imaging Experiments

Hela cells were seeded in each well of a confocal dish (a coverglass-bottom dish) and cultured at 37 °C for 24 h. An aqueous solution of the CDs (0.1 mg/mL) was passed through a 0.2 μm sterile membrane filter. The filtered fluorescent suspension (40, 50, and 60 μL) was mixed with the culture medium (200 μL) and then added to three wells of the confocal dish (the fourth used as a control) in which the Hela cells were grown. After an incubation period of 2 h, the medium was removed and the cells were washed thoroughly three times with phosphate buffered saline (PBS) and kept in PBS for the optical imaging. Cellular uptake of CDs by Hela cells was monitored by confocal microscopy under the excitation wavelength of 405 nm.

## 4. Conclusions

In conclusion, we have demonstrated that a fast, efficient and green method to synthesize fluorescent carbon dots by the hydrothermal treatment of lignin under the action of H_2_O_2_. The resulting CDs were thoroughly characterized and showed excellent potential for applications in biological labeling and biosensors fields.

## Abbreviations

The following abbreviations are used in this manuscript:

CDs: carbon dots
PL: photoluminescence
TEM: transmission electron microscopy
XPS: X-ray photo-electron spectroscopy
PBS: phosphate buffered saline
